# Estradiol decreases the excitability of RA projection neurons in adult male zebra finches

**DOI:** 10.3389/fncel.2023.1046984

**Published:** 2023-02-14

**Authors:** Yutao Zhang, Yalun Sun, Yanran Wu, Wei Sun, Kun Zhang, Wei Meng, Songhua Wang

**Affiliations:** ^1^Jiangxi Key Laboratory of Organic Chemistry, Jiangxi Science and Technology Normal University, Nanchang, China; ^2^School of Life Science, Jiangxi Science and Technology Normal University, Nanchang, China

**Keywords:** estradiol, excitability, robust nucleus of the arcopallium, zebra finches, projection neuron

## Abstract

Zebra finches are essential animal models for studying learned vocal signals. The robust nucleus of the arcopallium (RA) plays an important role in regulating singing behavior. Our previous study showed that castration inhibited the electrophysiological activity of RA projection neurons (PNs) in male zebra finches, demonstrating that testosterone modulates the excitability of RA PNs. Testosterone can be converted into estradiol (E2) in the brain through aromatase; however, the physiological functions of E2 in RA are still unknown. This study aimed to investigate the electrophysiological activities of E2 on the RA PNs of male zebra finches through patch-clamp recording. E2 rapidly decreased the rate of evoked and spontaneous action potentials (APs) of RA PNs, hyperpolarized the resting membrane potential, and decreased the membrane input resistance. Moreover, the G-protein–coupled membrane-bound estrogen receptor (GPER) agonist G1 decreased both the evoked and spontaneous APs of RA PNs. Furthermore, the GPER antagonist G15 had no effect on the evoked and spontaneous APs of RA PNs; E2 and G15 together also had no effect on the evoked and spontaneous APs of RA PNs. These findings suggested that E2 rapidly decreased the excitability of RA PNs and its binding to GPER suppressed the excitability of RA PNs. These pieces of evidence helped us fully understand the principle of E2 signal mediation *via* its receptors to modulate the excitability of RA PNs in songbirds.

## Introduction

17 β-Estradiol (E2), an endogenous estrogen, has been implicated in influencing behaviors. E2 directly acts on neuronal membranes to quickly influence brain function in rodents and songbirds (Balthazart and Ball, 2006; Tozzi et al., 2020; Zhang et al., 2021). E2 is synthesized from androgens, such as testosterone, by the enzyme aromatase. Estrogens can rapidly modulate the electrophysiological activity of different types of neurons (Kelly and Rønnekleiv, 2002). ERα and G-protein–coupled membrane-bound estrogen receptor (GPER) were found to be widespread, nonuniform, and overlapped with song control nuclei (Jacobs et al., 1999; Acharya and Veney, 2012). ERα was mainly expressed in HVC (used as a proper name). However, GPER was largely expressed in HVC and arcopallium (RA) (Acharya and Veney, 2012). These studies hinted that estrogens might quickly bind to GPER and thus affect RA.

In songbirds, E2 shapes auditory circuits to support communication learning and perception (Vahaba and Remage-Healey, 2015, 2018; Vahaba et al., 2020; Scarpa et al., 2022). Injection of fadrozole (an aromatase inhibitor) in zebra finches reduced the motivation to sing and song acoustic stereotypy on the same day, indicating that estrogens significantly affect the ability to modulate the singing behavior (Alward et al., 2016). The RA activity is significantly correlated with variations in the acoustic stereotypy of syllables (Sober et al., 2008). Therefore, RA plays an important role in regulating singing behavior. E2 has been demonstrated to be involved in adult song production (Alward et al., 2016). It is still unknown whether E2 modulates the electrophysiological activity of RA in adult male zebra finches to regulate the singing behavior. Therefore, we studied the roles of E2 in the excitability of RA projection neurons (PNs) using whole-cell patch-clamp recording in adult male zebra finches to shed light on this unsolved issue.

## Materials and methods

### Animals used in this study and preparation of brain slices

All experiments were performed on adult male zebra finches (*Taeniopygia guttata*) (>120 days old), which were obtained from a local breeder (*n* = 45). The methods of using zebra finches were authorized by the Institutional Animal Care and Use Committee of Jiangxi Science and Technology Normal University (3601020137931). Then, 300-μm-thick coronal brain slices were obtained from adult male zebra finches (Wang et al., 2020). The bird was anesthetized with isoflurane and decapitated. The brain was then placed in an ice-cold slice solution and oxygenated (5% CO_2_ and 95% O_2_). The slice solution was composed of 5 mM KCl, 28 mM NaHCO_3_, 248 mM sucrose, 1.3 mM MgSO_4_·7H_2_O, 10 mM glucose, and 1.26 mM NaH_2_PO_4_·H_2_O. The brain slices were sectioned using a vibrating microtome (700 sms; Campden Instruments, London, UK). The slices were transferred to a holding chamber containing oxygenated artificial cerebrospinal fluid (ACSF) at 35°C. The ACSF was composed of 125 mM NaCl, 2.5 mM KCl, 1.2 mM MgSO_4_·7H_2_O, 1.27 mM NaH_2_PO_4_·H_2_O, 25 mM NaHCO_3_, 25 mM glucose, and 2.0 mM CaCl_2_ (Meng et al., 2016). The slices were incubated for at least 0.5 h and equilibrated to room temperature prior to electrophysiological recordings.

### Electrophysiological recordings

At this stage, the brain slices were placed in a recording chamber under a BX51WI microscope (Olympus, Tokyo, Japan) equipped with an IR-DIC video camera, having 10 × and 40 × lenses with optical zoom and superfused with oxygenated ACSF (Proano and Meitzen, 2020). Recording pipettes were fabricated using borosilicate glass *via* a Flaming–Brown puller and then filled with the intercellular solution comprising 5 mM NaCl, 120 mM KMeSO_4_, 2 mM EGTA, 10 mM HEPES, 2 mM ATP, and 0.3 mM GTP (pH 7.2–7.4, 340 mOsm). The recording pipettes (with a resistance of 4–7 MΩ) were positioned using an integrated motorized control system. Whole-cell recordings were performed using standard techniques. The two cell types in the RA, namely, PNs and GABAergic interneurons, were identified based on their distinct electrophysiological properties (Meng et al., 2016). The junction potentials were modified before recording the PNs. The pipette capacitance and series resistance were quickly compensated using MultiClamp 700B (Molecular Devices, CA, USA), which was monitored at 2-min intervals. The signals were amplified, filtered (2 kHz), and digitized (10 kHz) using a MultiClamp 700B amplifier attached to a Digidata 1550 system and a computer using the pCLAMP version 10.7 software (Wang et al., 2020). The membrane potentials were corrected for a liquid junction potential of +5 mV. The recordings that showed series resistance >20 MΩ or 10% change were excluded from the analysis. The signals of recording neurons were allowed to stabilize at 3–5 min after the whole-cell clamp.

### Drug application

Previous studies used E2 to investigate how estrogen rapidly regulates neuronal activities (Smejkalova and Woolley, 2010; Krentzel et al., 2018; Tozzi et al., 2020). G1 is an agonist and G15 is an antagonist for GPER in rodents in rodents and zebra finches, respectively (Bologa et al., 2006; Blasko et al., 2009; Bailey et al., 2017; Tehrani and Veney, 2018). We mixed E2 with DMSO to form a 1-mM stock solution and then diluted the stock with ACSF to obtain 100 pM E2. Then, G1/G15 was mixed with DMSO to form a 1-mM stock solution, and the stock solution was diluted using ACSF 100 times to obtain 10 μM G1 (Kim et al., 2016; Krentzel et al., 2018), or the stock was diluted with ACSF 10,000 times to obtain 100 pM G1/G15. G1 is a selective agonist for GPER that does not bind ERα and ERβ at concentrations up to 10 μM *in vitro* (Bologa et al., 2006; Blasko et al., 2009). For avoiding the issues of off-target of G1 effects on the neurons, 10 μM and 100 pM were selected as the concentration of G1. All drugs were obtained from Sigma-Aldrich, MO, USA.

### Data analysis

Data were obtained using pCLAMP 10.7 (Molecular Devices, CA, USA) *via* a Digidata 1550B series A/D board (Molecular Devices, CA, USA) at a sampling frequency of 10 kHz. Data from action potential (AP) were analyzed using pCLAMP 10.7 and Origin Pro 8.0 (OriginLab Corporation, USA) on a computer. The AP threshold was detected using a custom algorithm described previously by Baufreton et al. (2005). The half-width of AP was measured as a duration between 50% rise time and 50% decay time (measured from baseline) of APs (Rodríguez-Molina et al., 2014). The afterhyperpolarization (AHP) peak amplitude was the difference between the AP threshold and the most negative voltage reached during the AHP. The AHP time-to-peak was the time of this minimum minus the time when the membrane potential crossed the AP threshold on the descent from the AP peak (Farries et al., 2005). For obtaining data on each neuron, the current of the same intensity (100 pA with 5 ms duration, with an interval time of 1 min) was injected five times to induce a single AP in the pre-drug and the steady state of drug effects and then the average value of the intrinsic electrophysiological properties of these five APs was taken as the final value. The membrane time constant, membrane input resistance, and the slope of the current–voltage curve were calculated as described in our previous study (Wang et al., 2014). The data were presented as the means ± SEM and compared using paired two-tailed Student's *t*-tests (*P* < 0.05 indicated a statistically significant difference), except as otherwise noted.

## Results

Stable whole-cell recordings were obtained using 145 RA PNs from 89 slices of 45 male zebra finches.

### E2 affected the excitability of RA PNs in a concentration-dependent manner

**Figure 1:**
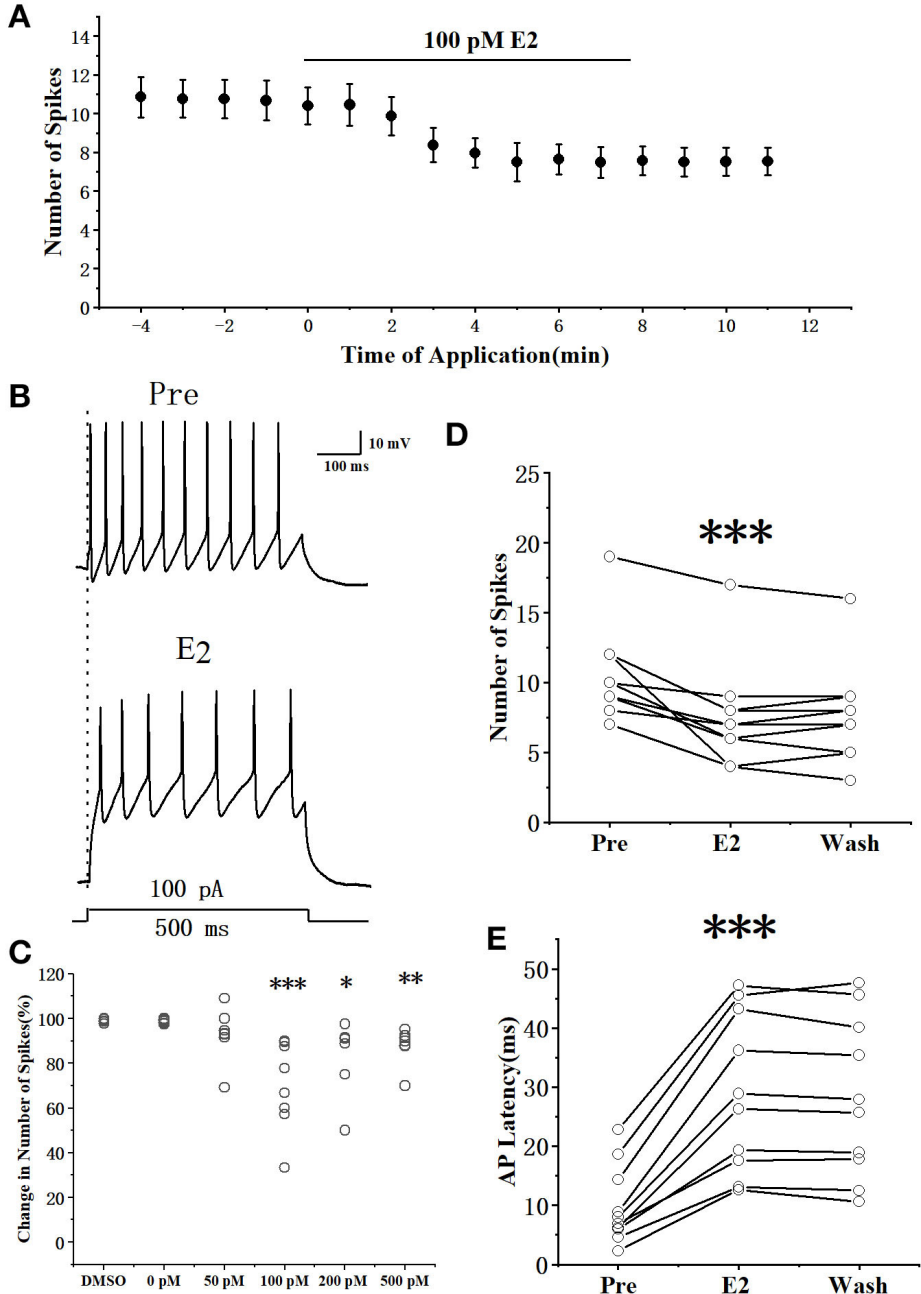
E2 reduced the evoked AP firing of RA PNs. **(A)** Number of spikes in response to 100 pA current for 500 ms before and during the application of E2 (*n* = 12). **(B)** Example traces from the experiment shown in **(A)**. **(C)** Statistical diagram of 0.1% DMSO and different concentrations of E2 (0, 50, 100, 200, and 500 pM) depicting the change in the number of spikes at the steady state compared with that at the pre-drug stage. **(D)** Evoked spikes significantly decreased in the presence of E2 by depolarizing step pulse (*n* = 12). **(E)** Evoked AP latency increased in the presence of E2 (*n* = 12). *P < 0.05, **P < 0.01, ***P < 0.001. The temperature used during electrophysiological recordings was 16–20°C.

The suprathreshold currents (100 pA with 500 ms duration and 1-min interval) were injected into the RA PNs to test the effect of E2 on AP generation (Figures 1A, B). As shown in Figure 1C, the application of E2 at concentrations of 100 pM or above affected evoked AP. The number of evoked AP showed a concentration-dependent decrement (a repeated one-way ANOVA, 0 pM vs. 50 pM: *F*_(1, 12)_ = 1.62, *P* = 0.23;0 pM vs. 100 pM: *F*_(1, 17)_ = 17.71, *P* < 0.001; 0 pM vs. 200 pM: *F*_(1, 12)_ = 6.46, *P* = 0.026; 0 pM vs. 200 pM: *F*_(1, 12)_ = 12.38, *P* < 0.01). As E2 dissolved in DMSO, we considered DMSO (0.1% in ACSF) as the control group to verify whether E2 has an effect on RA PNs. As shown in Figure 1C, DMSO had no effect on the excitability of RA PNs (the percentage change in the number of spikes compared with the pre-drug was 99.15 ± 0.37%, *n* = 6). The application of 100 pM E2 significantly decreased the number of spikes, reaching a steady state after 6 min of E2 application (Figure 1A). The application of 100 pM E2 significantly decreased the number of spikes from 10.42 ± 0.96 to 7.50 ± 1.00 (*n* = 12; *P* < 0.001, *t* = 5.12) (Figure 1D). This effect was not reversible (Smejkalova and Woolley, 2010). Moreover, 100 pM E2 increased the evoked AP latency from 9.86 ± 2.19 to 28.99 ± 4.45 ms (*n* = 12, *P* < 0.01, *t* = 7.74; Figure 1E), suggesting that E2 decreased the excitability of RA PNs. As 100 pM E2 suppressed to the maximum extent of the excitability of RA PNs, this concentration was adopted for subsequent experiments in this study.

**Figure 2:**
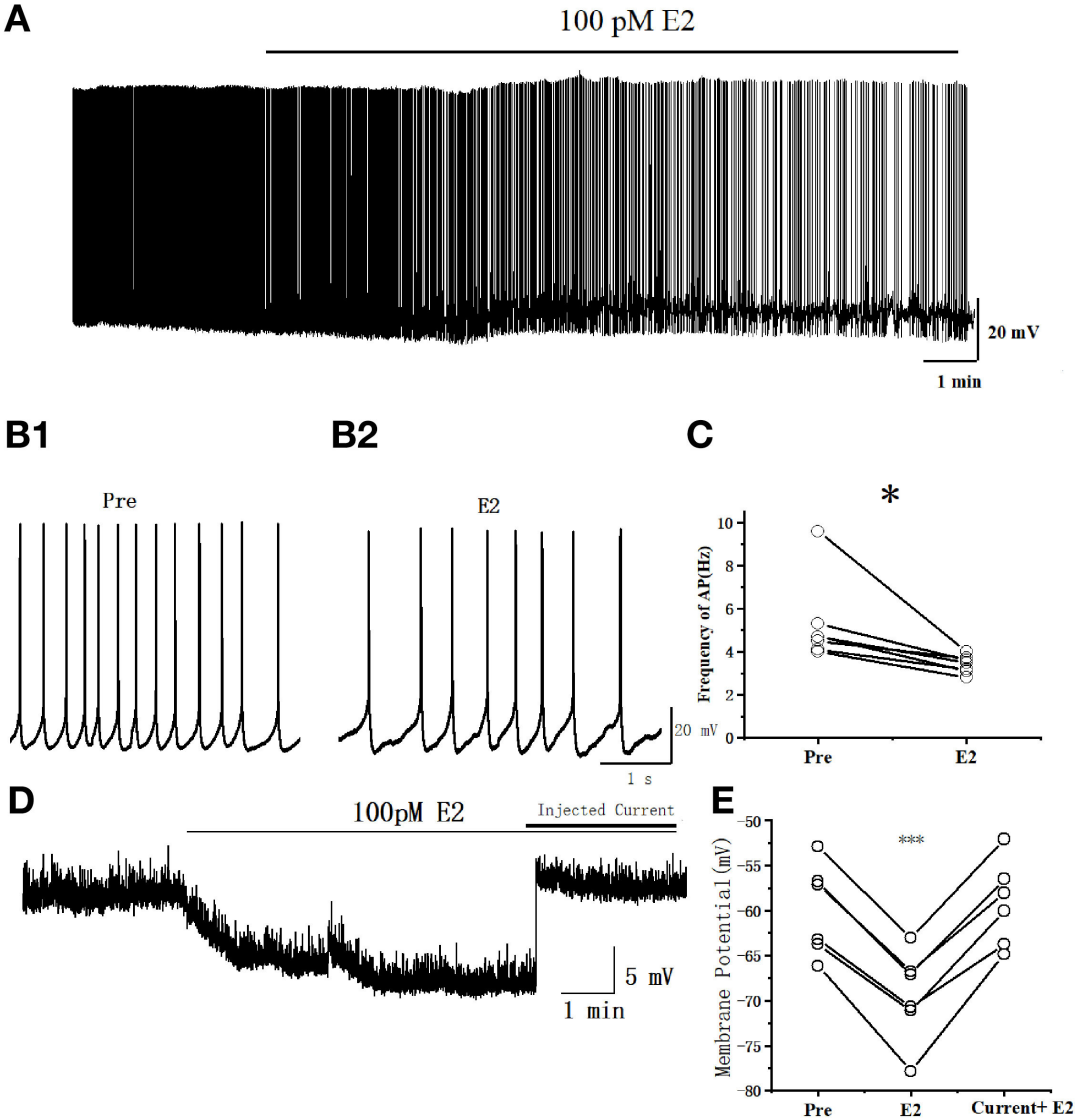
E2 decreased the spontaneous AP firing frequency in RA PNs. **(A)** A representative whole-cell recording showing the effects of 100 pM E2 on the spontaneous AP firing frequency. **(B1, B2)** Enlarged example trace of AP firing before **(B1)** and during **(B2)** E2 application from **(A)**. **(C)** Inhibitory effects of E2 on the spontaneous AP firing frequency (*n* = 7). *P < 0.05. **(D)** Representative whole-cell recording showing the effects of 100 pM E2 and injected current on the resting membrane potential. **(E)** Resting membrane potentials were hyperpolarized during the application of E2 (*P* < 0.001, *n* = 6) and then the cell returned to its excitability state at the pre-drug stage when given a steady-state depolarizing current injection (20–50 pA) (*P* = 0.30, *n* = 6). ***P < 0.001.

The spontaneous AP of RA PNs was recorded with the conventional whole-cell patch recording under current–clamp configurations to examine further the effects of E2 on the excitability of RA PNs. The application of 100 pM E2 significantly decreased the spontaneous AP firing frequency from 5.27 ± 0.80 to 3.41 ± 0.17 Hz (*n* = 7, *P* = 0.027, *t* = 2.92) (Figure 2). These results indicated that E2 inhibited the excitability of RA PNs.

We added 1 μM tetrodotoxin (TTX) to test whether the changing of resting membrane potential leads to a reduction in the excitability of RA PNs by E2. Indeed, the resting membrane potential was hyperpolarized during the application of E2 (Pre: −59.96 ± 2.29 mV, E2: −69.42 ± 2.28 mV, *P* < 0.001, *n* = 6, *t* = 13.67; Figures 2D, E), reaching to a steady state after 5 min of E2 application and then returning to the cell to its excitability potential of pre-drug when given a steady-state depolarizing current injection (Pre: 59.96 ± 2.29 mV, current + E2: −59.18 ± 2.12 mV, *P* = 0.30, *n* = 6, *t* = 1.15; Figures 2D, E). The amount of current necessary to return cells to their original membrane potential following E2 exposure depended on the initial potential (the current was 20–50 pA). These results demonstrated that E2 acts through hyperpolarizing resting membrane potential to decrease the excitability of RA PNs.

### E2 affected the intrinsic electrophysiological properties of RA PNs

**Figure 3:**
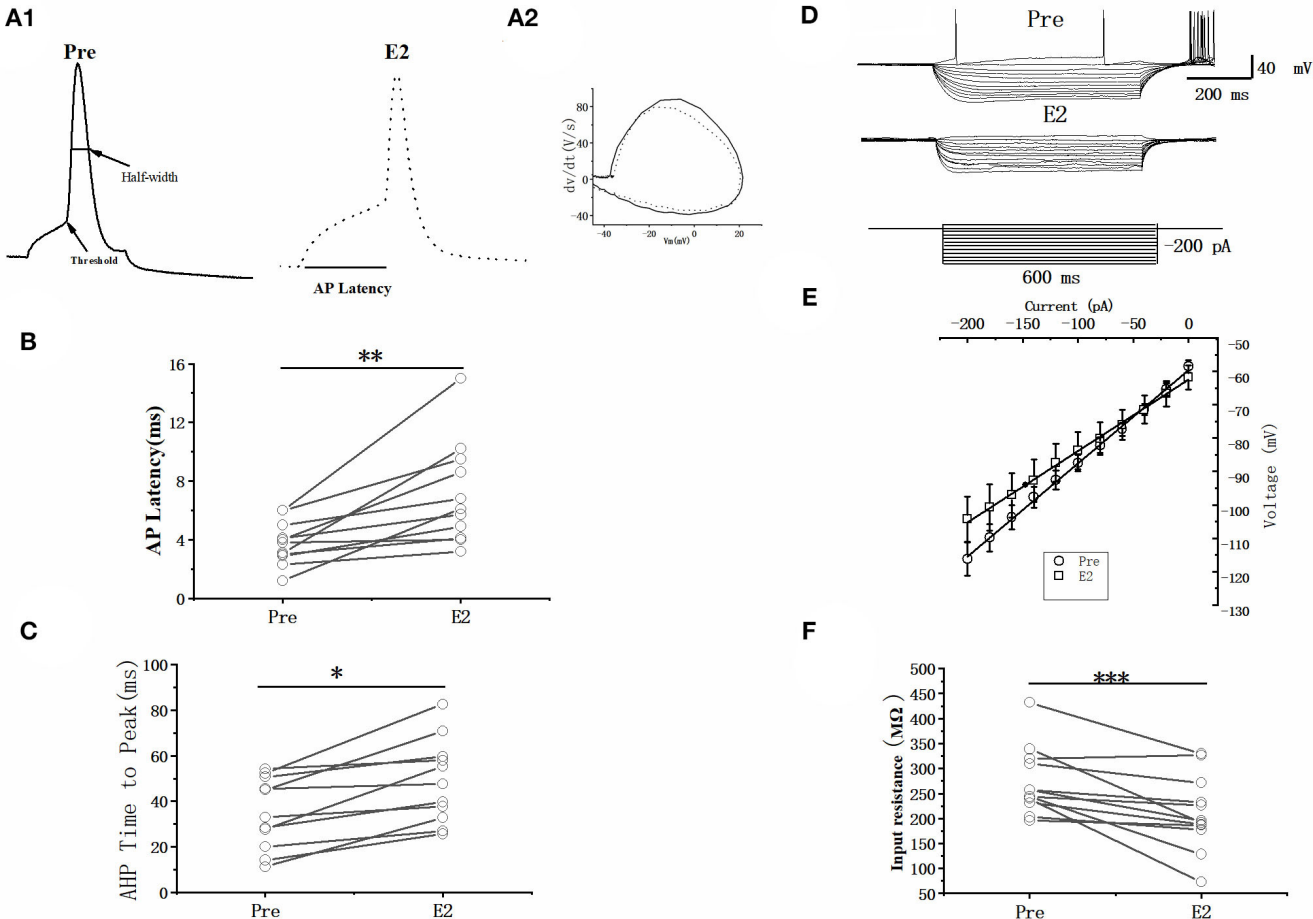
Effects of E2 (100 pM) on the intrinsic electrophysiological properties of RA PNs. **(A1)** Sample of single AP evoked in response to 100 pA current for 5 ms before and during the application of E2. **(A2)** The derivatives of AP in A1. **(B)** AP latency significantly prolonged in the presence of E2 (*n* = 11). **(C)** AHP time-to-peak decreased significantly in the presence of E2 (*n* = 11). **(D)** Voltage responses of a neuron to a series of hyperpolarizing current steps before, during, and after the application of E2. **(E)** Current–voltage curves of pre and E2-treated stages (*n* = 11). **(F)** Membrane input resistance decreased in the presence of E2 (*n* = 13). *P < 0.05, **P < 0.01, ***P < 0.001.

As can be seen in the schematic presented in Figure 3A, we used a current pulse of 100 pA at 5 ms to test the change in the intrinsic electrophysiological properties of RA PNs after the application of 100 pM E2. E2 increased the evoked AP latency from 3.76 ± 0.47 to 7.10 ± 1.10 ms (*n* = 11, *P* < 0.01, *t* = 3.96; Figure 3B and Table 1). The AHP time-to-peak was prolonged (Pre: 34.72 ± 4.95 ms, E2: 48.77 ± 5.78 ms, *P* < 0.01, *n* = 11, *t* = 4.51; Figure 3C and Table 1), and the AHP peak amplitude was increased (Pre: −20.62 ± 0.49 mV, E2: −22.79 ± 0.70 mV, *P* = 0.01, *n* = 11, *t* = 3.01, DF = 10) during the application of E2 (Table 1). The AP threshold, half-width, and peak amplitude were unaffected (Table 1). Two cells exposed to E2 could not induce a single AP because a short-duration 5-ms pulse would not always lead to an evoked AP of RA PNs. Moreover, the effect of E2 on the membrane input resistance of RA PNs was also recorded. As shown in Table 1 and Figure 3D to F, the membrane input resistance decreased during the application of E2 (Pre: 271.44 ± 18.53 MΩ, E2: 209.50 ± 20.55 MΩ, *P* < 0.01, *n* = 13, *t* = 4.07). These results demonstrated that E2 decreased the membrane input resistance to inhibit the excitability of RA PNs.

**Table 1:** Intrinsic properties of RA PNs before and during E2 application.

**Parameter**	**Pre-drug stage**	**E2 application stage**	***t*-value*, P*-value**
AP threshold (mV, *n =* 11)	−41.06 ± 1.63	−38.91 ± 3.00	*t =*−0.77, *P* = 0.46
AP latency (ms, *n =* 11)	3.76 ± 0.47	7.10 ± 1.10^**^	*t =* −3.96, *P* = 0.003
Peak amplitude (mV, *n =* 11)	83.84 ± 4.33	80.15 ± 5.47	*t =* −0.78, *P* = 0.46
Half-width (ms, *n =* 11)	2.06 ± 0.22	2.65 ± 0.44	*t =* −1.40, *P* = 0.19
AHP peak amplitude (mV, *n =* 11)	−20.62 ± 0.49	−22.79 ± 0.70^*^	*t =* 3.01, *P* = 0.01
AHP time-to-peak (ms, *n =* 11)	34.72 ± 4.95	48.77 ± 5.78^**^	*t* =-4.51, *P* = 0.001
Membrane input resistance (MΩ, *n =* 13)	271.44 ± 18.53	209.50 ± 20.55^**^	*t* =4.07, *P* = 0.002

^*^P < 0.05, ^**^P < 0.01.

### GPER agonist decreased the excitability of RA PNs

**Figure 4:**
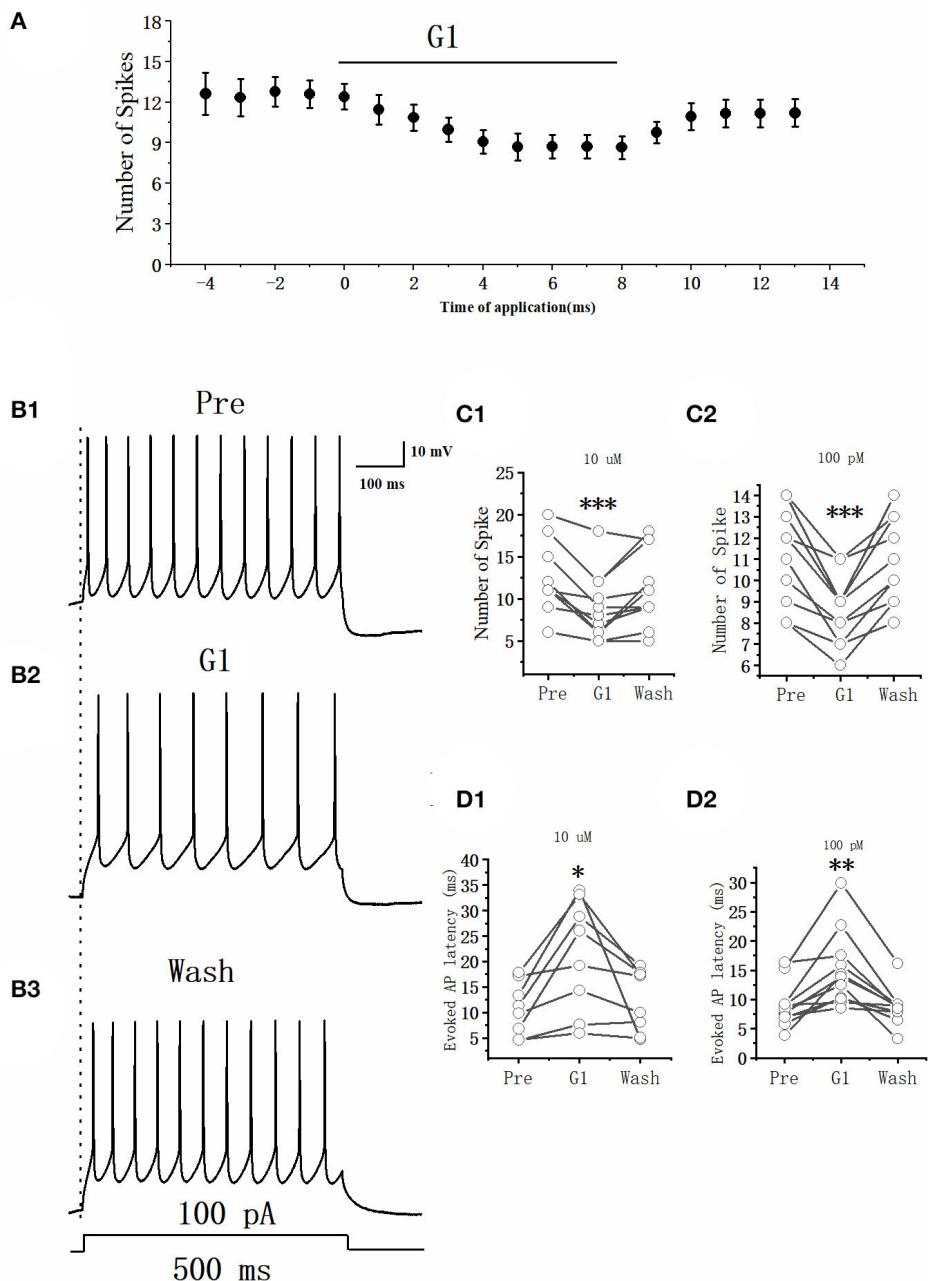
Effect of G1 (10 μM) on the excitability of RA PNs. **(A)** Time course of the number of evoked spikes in the presence of G1. The line at the top indicates that G1 (10 μM) was present in the bath. **(B1–B3)** Example traces from the experiment shown in **(A)**. **(C1)** Statistical scatterplot of the number of evoked spikes at the pre, 10 μM G1-treated, and washout stages (*n* = 13). **(C2)** Statistical scatterplot of the number of evoked spikes at the 100 pM G1-treated stage (*n* = 11). **(D1)** Statistical scatterplot of evoked AP latency at the pre, 10 μM G1-treated, and washout stages (*n* = 13). **(D2)** Statistical scatterplot of evoked AP latency at the 100 pM G1-treated stage (*n* = 11). *P < 0.05, **P < 0.01, ***P < 0.001.

As E2 rapidly suppressed the excitability of RA PNs, whether the E2-binding GPER would modulate the excitability of RA PNs needed further exploration. This study used the GPER agonist G1 (10 μM, 100 pM) to determine its effects. The effect of G1 on the evoked AP of RA PNs with 100 pA currents and 500 ms duration was examined (Figures 4A, B). The application of G1 significantly decreased the number of spikes of RA PNs, reaching a steady state after 6 min of application. As shown in Figure 4C1, the number of spikes was suppressed after an application of 10 μM G1 (Pre: 12.62 ± 1.27, G1: 8.69 ± 1.06, *n* = 13, *P* < 0.001, *t* = 6.08) and returned to 11.15 ± 1.17 (*n* = 13) after G1 was washed for 3 min. As shown in Figure 4C2, the number of spikes was suppressed after the application of 100 pM G1 (Pre: 11.36 ± 0.74, G1: 8.55 ± 0.78, *n* = 11, *P* < 0.001, *t* = 6.08) and returned to 11.40 ± 1.13 (*n* = 11) after G1 was washed for 3 min. Furthermore, G1 markedly increased the evoked AP latency (Pre: 10.80 ± 2.12 ms, 10 μM G1: 22.09 ± 4.37 ms, *n* = 13, *P* < 0.05, *t* = 3.09; Pre: 8.98 ± 1.13 ms, 100 pM G1: 15.01 ± 3.21 ms, *n* = 11, *P* < 0.01, *t* = 4.07) (Figures 4D1, D2), indicating that G1 suppressed the excitability of RA PNs.

**Figure 5:**
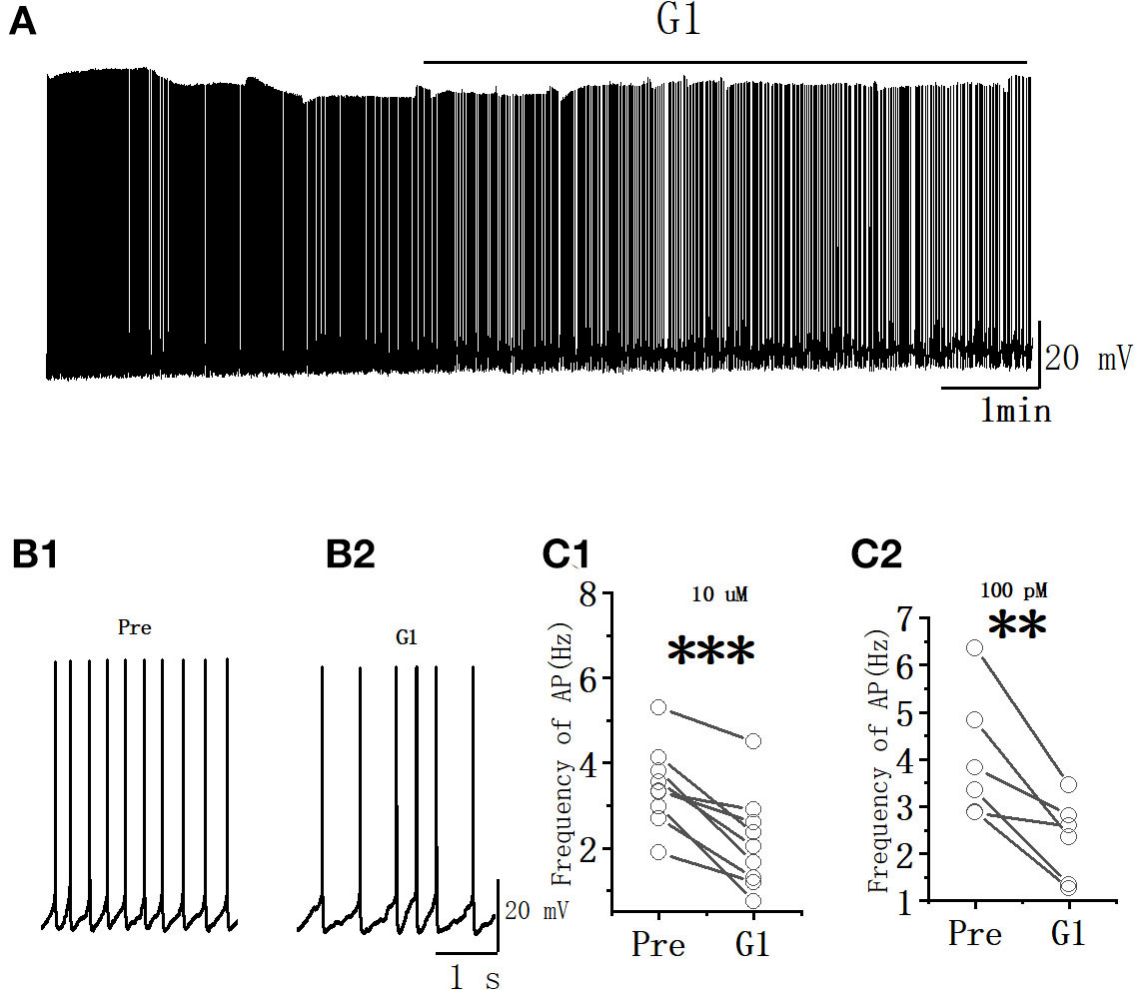
G1 decreased the spontaneous AP firing frequency in RA PNs. **(A)** Representative whole-cell recording showing the effects of G1 (10 μM) on the spontaneous AP firing frequency. **(B1, B2)** Enlarged example trace of AP firing before **(B1)** and during **(B2)** the application of G1 from **(A)**. **(C1)** Statistical scatterplot of the frequency of AP in the presence of 10 μM G1 (*n* = 9). **(C2)** Statistical scatterplot of the frequency of AP in the presence of 100 pM G1 (*n* = 6). ^**^P < 0.01 and ^***^P < 0.001.

The spontaneous AP of RA PNs was tested to examine further the effects of G1 on the excitability of RA PNs. As shown in Figure 5, the application of G1 significantly decreased the spontaneous AP firing frequency (10 μM G1: 3.45 ± 0.33 to 2.15 ± 0.40 Hz, *n* = 9, *P* < 0.001, *t* = 5.85; Figure 5C1; 100 pM G1: 4.02 ± 0.61 to 2.30 ± 0.38 Hz, *n* = 6, *P* < 0.01, *t* = 4.36; Figure 5C2). These results indicated that G1 of two different concentrations inhibited the excitability of RA PNs.

### GPER agonist affected the intrinsic electrophysiological properties of RA PNs

**Figure 6:**
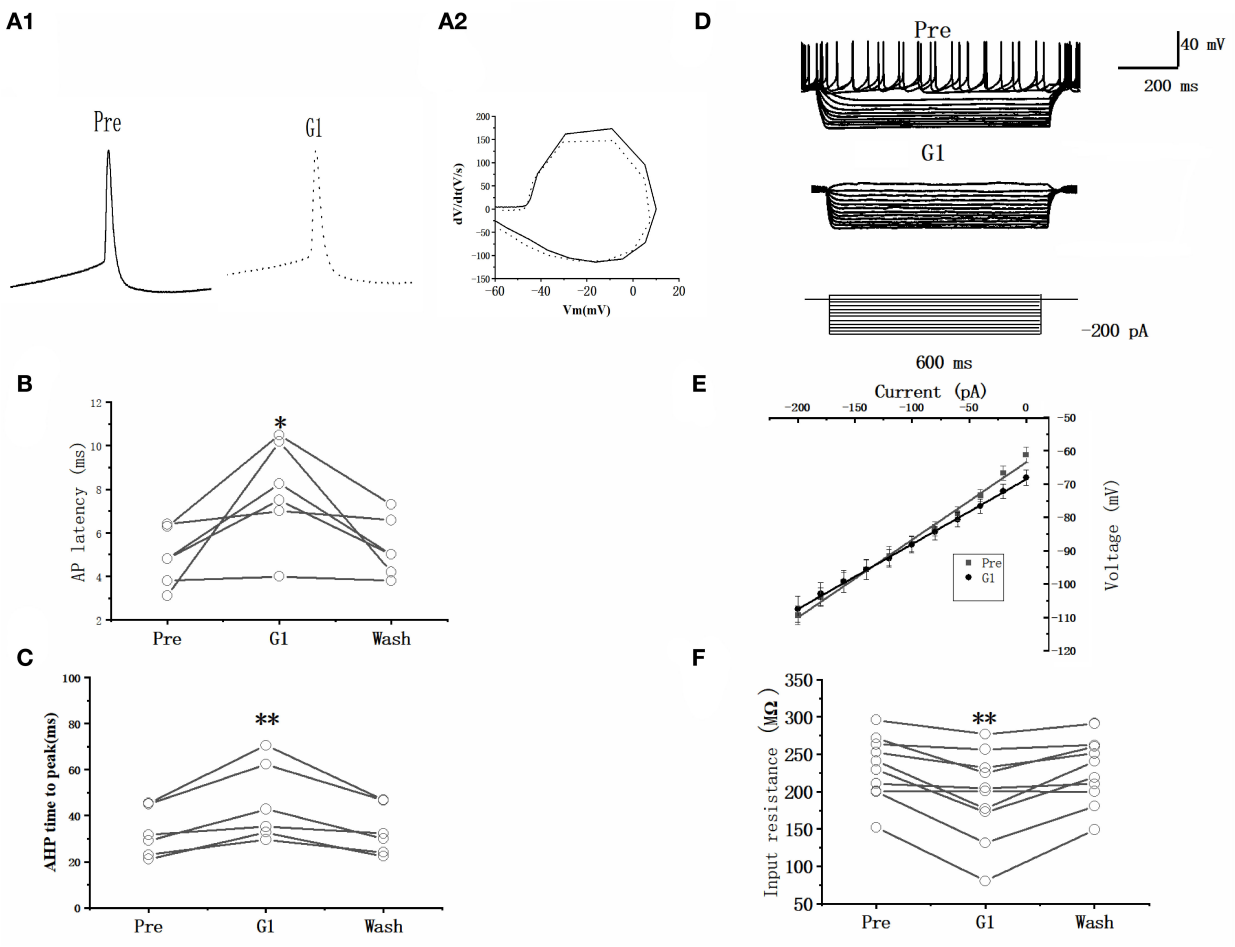
Effect of G1 (10 μM) on the intrinsic electrophysiological properties of RA PNs. **(A1)** Representative AP recordings in response to a depolarizing pulse of 100 pA for 5 ms at the pre, G1-treated, and washout stages. **(A2)** The derivatives of AP in A1. **(B)** AP latency significantly decreased in the presence of G1 (*n* = 6). **(C)** AHP time-to-peak significantly decreased in the presence of G1. **(D)** Voltage responses of a neuron to a series of hyperpolarizing current steps before, during, and after the application of G1 (*n* = 6). **(E)** Current–voltage curves during the application of G1 (*n* = 6). **(F)** Statistical scatterplot of the membrane input resistance at the pre, G1-treated, and washout stages (*n* = 11). ^*^P < 0.05 and **P < 0.01.

A current pulse of 100 pA at 5 ms was used to explore the role of G1 on the intrinsic electrophysiological properties of RA PNs (Figure 6A). The application of 10 μM G1 increased the evoked AP latency from 4.87 ± 0.59 to 7.91 ± 1.07 ms (*n* = 6, *P* = 0.03, *t* = 2.94; Figure 6B and Table 2). The AHP time-to-peak prolonged after the application of G1 (Pre: 32.59 ± 4.72 ms, G1: 45.55 ± 7.59 ms, *P* < 0.01, *n* = 6, *t* = 4.11; Table 2 and Figure 6C), and the AHP peak amplitude increased (Pre: −21.81 ± 0.82 mV, G1: −26.61 ± 1.05, *P* < 0.01, *n* = 6, *t* = 4.58; Table 2). However, the AP threshold, half-width, and peak amplitude were uninfluenced (Table 2). Moreover, the effect of G1 on the membrane input resistance of RA PNs was also explored. G1 suppressed the membrane input resistance and returned to the control level after G1 washout (Pre: 237.50 ± 14.05 MΩ, G1: 203.10 ± 19.23 MΩ, *P* < 0.01, *n* = 11, *t* = 4.17; Table 2 and Figures 6D–F). These results indicated that activating GPER affected the intrinsic electrophysiological properties of RA PNs.

**Table 2:** Intrinsic properties of RA PNs before and during the application of G1.

**Parameter**	**Pre-drug stage**	**G1 application stage**	***t*-value, *P*-value**
AP threshold (mV, *n =* 6)	−40.35 ± 1.97	−42.03 ± 5.16	*t* =0.46, *P* = 0.67
AP latency (ms, *n =* 6)	4.87 ± 0.59	7.91 ± 1.07^*^	*t* =-2.94, *P* = 0.03
Peak amplitude (mV, *n =* 6)	81.51 ± 7.23	88.46 ± 9.15	*t* =2.46, *P* = 0.03
Half-width (ms, *n =* 6)	1.49 ± 0.21	1.74 ± 0.40	*t* =-0.97, *P* = 0.37
AHP peak amplitude (mV, *n* =6)	−21.81 ± 0.82	−26.61 ± 1.05^**^	*t* =4.58, *P* =0.006
AHP time-to-peak (ms, *n =* 6)	32.59 ± 4.72	45.55 ± 7.59^**^	*t =* −4.11, *P* = 0.009
Membrane input resistance (MΩ, *n =* 11)	237.50 ± 14.05	203.10 ± 19.23^**^	*t =* 4.17, *P* = 0.002

^*^P < 0.05, ^**^P < 0.01.

### GPER antagonist had no effect on the excitability of RA PNs

**Figure 7:**
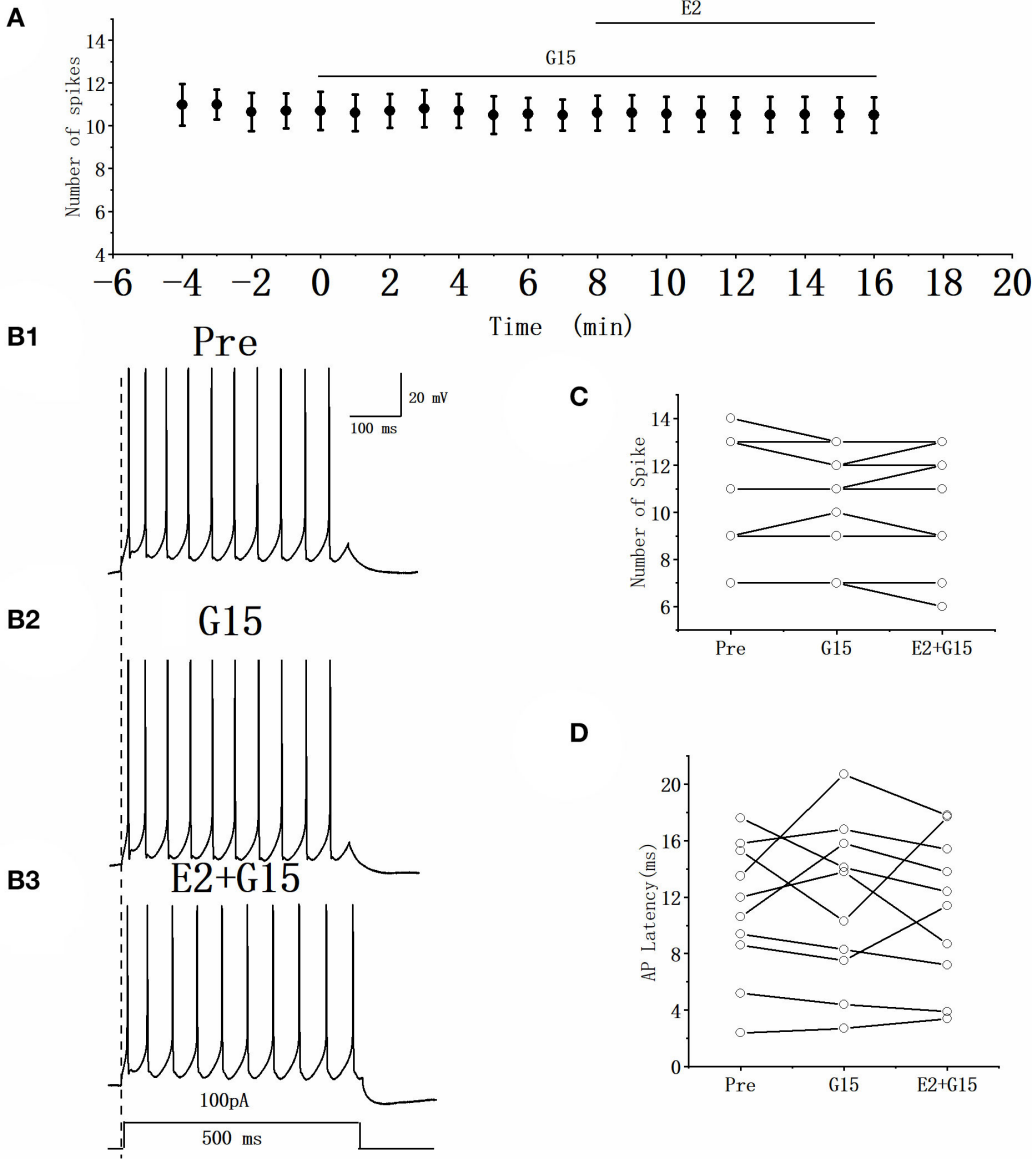
Effect of G15 (100 pM) and E2 (100 pM) + G15 (100 pM) on the excitability of RA PNs. **(A)** Time course of the number of evoked spikes in the presence of G15 and E2 + G15 (*n* = 10). The line at the top indicates the G15 and E2 + G15 were present in the bath. **(B1–B3)** Example traces from the experiment shown in **(A)**. **(C)** Statistical scatterplot of the number of evoked spikes in the presence of G15 and E2 + G15 (*n* = 10). **(D)** Statistical scatterplot of evoked AP latency in the presence of G15 and E2 + G15 (*n* = 10).

**Figure 8:**
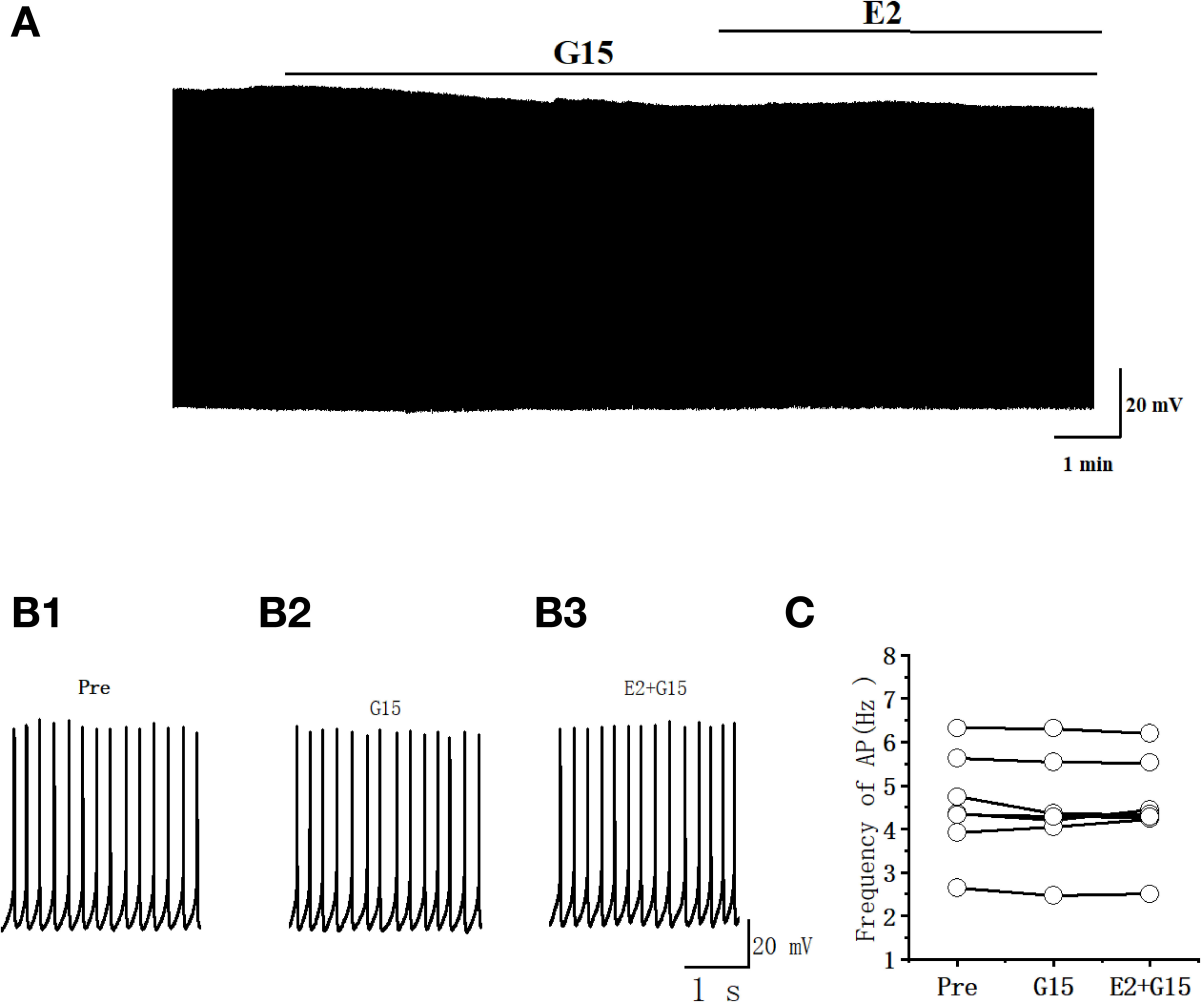
G15 and E2 + G15 had no effect on the spontaneous AP firing frequency in RA PNs. **(A)** Representative whole-cell recording showing the effects of G15 and E2 + G15 on the spontaneous AP firing frequency. **(B1, B2)** Enlarged example trace of AP firing before **(B1)** and during **(B3)** the application of G15 and E2 + G15 from **(A)**. **(C)** Statistical scatterplot of the frequency of AP in the presence of G15 and E2 + G15 (*n* = 7).

Whether the E2-binding GPER modulated the excitability of RA PN was further verified. We tested the actions of GPER antagonist G15 on the evoked AP firing of 100 pA for 500 ms and then added 100 pM E2 based on the concentration of G15 (Figures 7A, B). As high-concentration (10 μM) G1 and low-concentration (100 pM) G1 had similar effects on the excitability of RA PNs, 100 pM was selected as the final concentration of G15. As shown in Figure 7C, the application of 100 pM G15 at the steady state for 8 min had no effect on the number of evoked spikes compared with that at the “pre-drug” stage (Pre: 10.70 ± 0.86, G15: 10.50 ± 0.74, *P* = 0.34, *t* = 1.00, *n* = 10; Figure 7C), implying that the application of E2 and G15 for 8 min also had no effect on the number of evoked APs (Pre: 10.70 ± 0.86, E2 + G15: 10.50 ± 0.86; *P* = 0.34, *t* = 1.50; Figure 7C). Next, the evoked AP latency was analyzed. G15, E2, and G15 had no effect at the steady state on the evoked AP latency compared with that at the pre-drug stage (Pre: 11.04 ± 1.60 ms, G15: 11.44 ± 1.92 ms, *P* = 0.74, *t* = 0.34; E2 + G15: 11.17 ± 1.75 ms, *P* = 0.90, *t* = 0.13, *n* = 10 (Figure 7D). We also examined the effect of E2 and G15 on the spontaneous AP of RA PNs. As can be seen in Figure 8, no statistically significant difference was observed after the application of G15, E2, and G15 at the steady state compared with that at the pre-drug stage (Pre: 4.56 ± 0.49 Hz, G15: 4.45 ± 0.45 Hz, *P* = 0.14, *t* = 1.70; E2 +G15: 4.50 ± 0.44 Hz, *P* = 0.51, *t* = 0.70, *n* = 7). Hence, these results indicated that the E2-binding GPER modulated the excitability of RA PNs.

**Figure 9:**
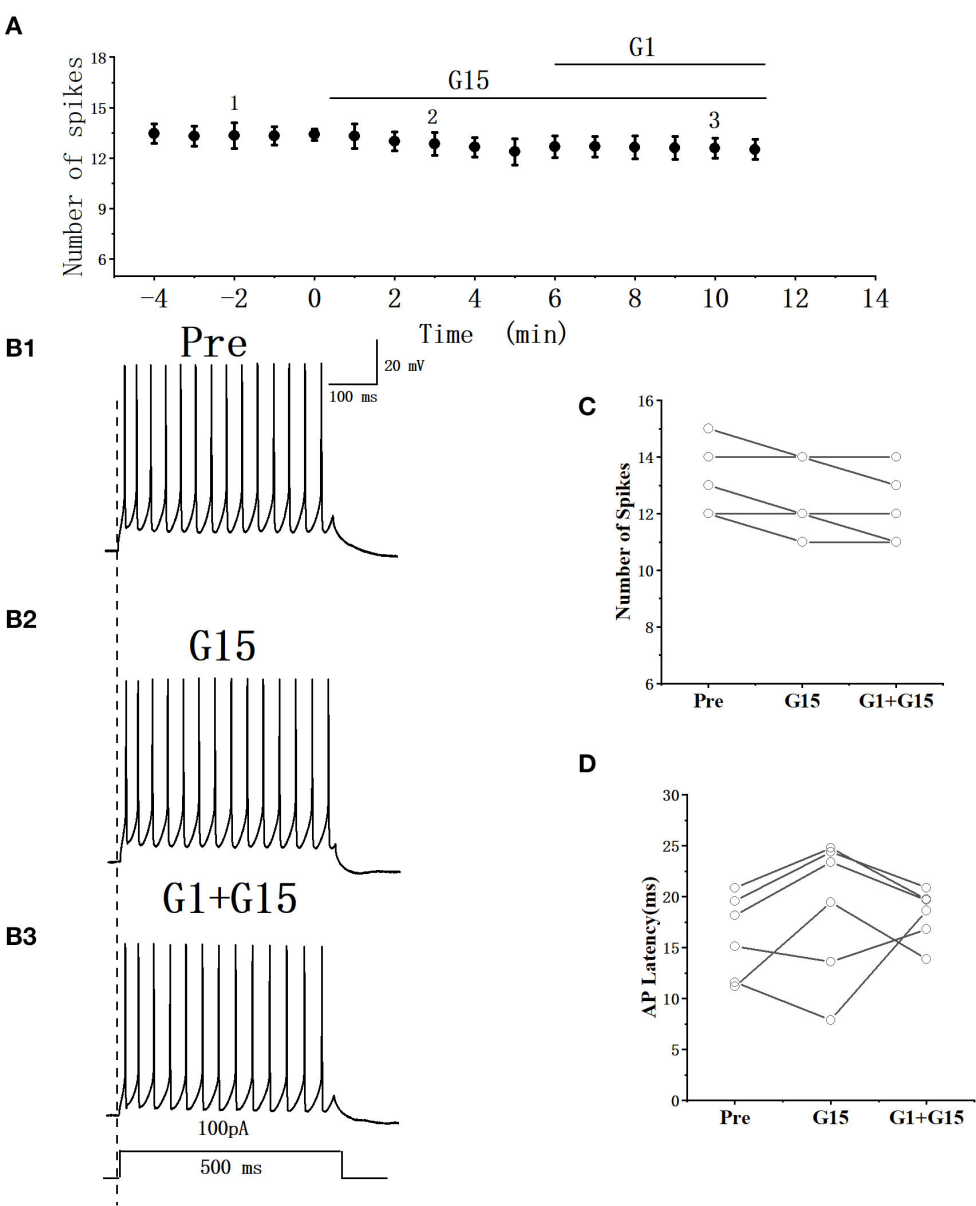
Effect of G1+ G15 on the excitability of RA PNs. **(A)** Time course of the number of evoked spikes in the presence of G1 + G15 (*n* = 6). The line at the top indicates the G1 + G15 present in the bath. **(B1–B3)** Example traces from the experiment shown in A. **(C)** Statistical scatterplot of the number of evoked spikes in the presence of G1+ G15 (*n* = 6). **(D)** Statistical scatterplot of evoked AP latency in the presence of G1 + G15.

**Figure 10:**
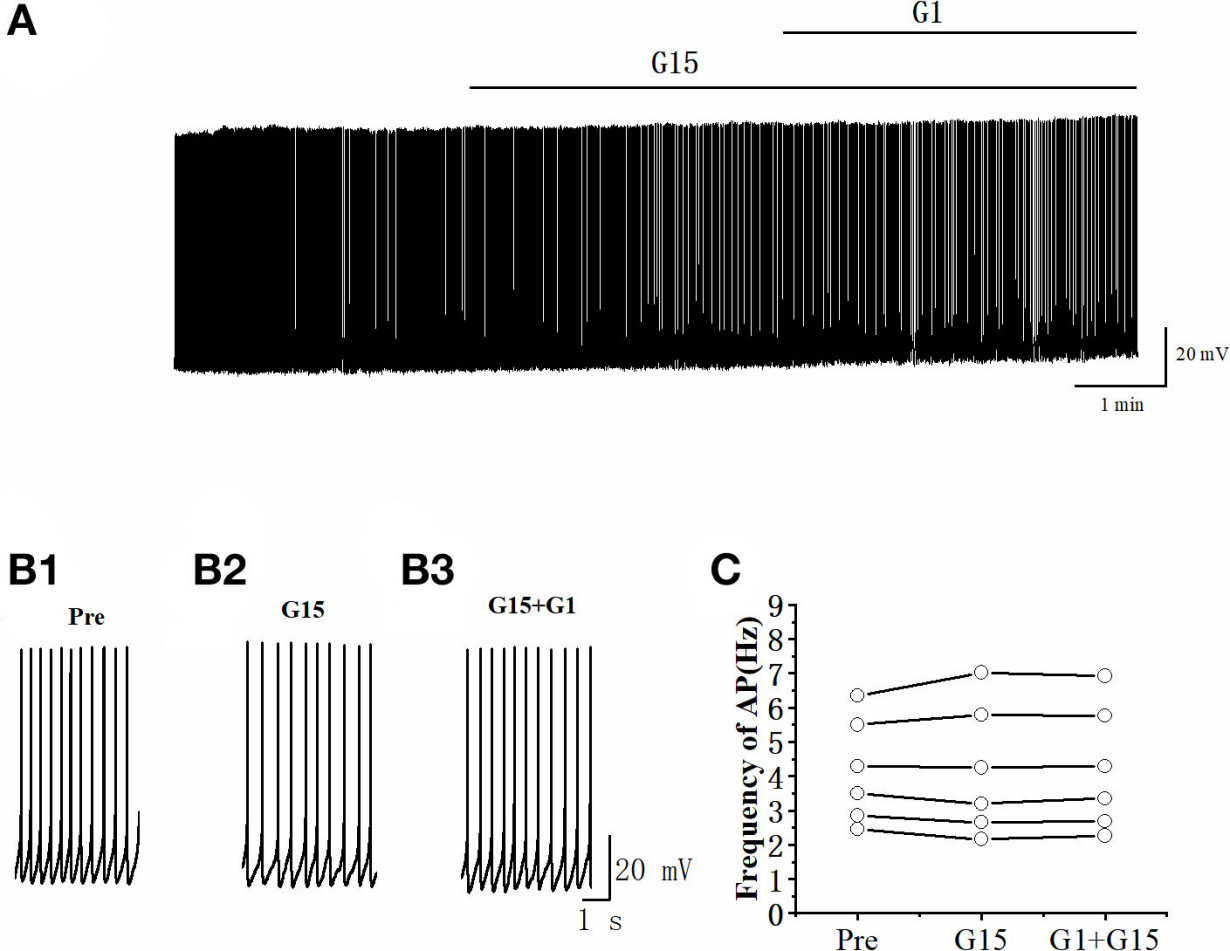
G1 + G15 had no effect on the spontaneous AP firing frequency in RA PNs. **(A)** Representative whole-cell recording showing the effect of G1 + G15 on the spontaneous AP firing frequency. **(B1–B3)** Enlarged example trace of AP firing before **(B1)**, during G15 **(B2)**, and G15 + G1 **(B3)** application of from **(A)**. **(C)** Effect of G1 + G15 on the spontaneous AP firing frequency (*n* = 6).

Whether G15 blocked the effects of G1 on RA PNs was further verified. As can be seen in Figures 9A, B, we tested the actions of G1 combined with G15 on the evoked AP firing by 100 pA at 500 ms duration. As shown in Figure 9C, G1 and G15 had no change in the number of evoked APs (Pre: 13.33 ± 0.54; G1 + G15: 12.83 ± 0.59; *P* = 0.08, *t* =2.24; wash: 12.50 ± 0.61; *n* = 6; Figure 9C). G1 and G15 had no effect on the evoked AP latency (Pre: 16.07 ± 1.84 ms; G1 + G15: 18.92 ± 3.06 ms; *P* = 0.18, *t* = 1.54; wash: 18.26 ± 1.14 ms; *n* = 6; Figure 9D). We also tested the effect of G1 and G15 on the spontaneous AP of RA PNs. As can be seen in Figure 10, there is also no significant difference after the application of G1 and G15 (Pre: 4.16 ± 0.68 Hz; G1 + G15: 4.18 ± 0.85 Hz; *n* = 6, *P* = 0.89, *t* = 0.14). Altogether, these results indicated that G15 blocked the effects of G1 on RA PNs.

**Figure 11:**
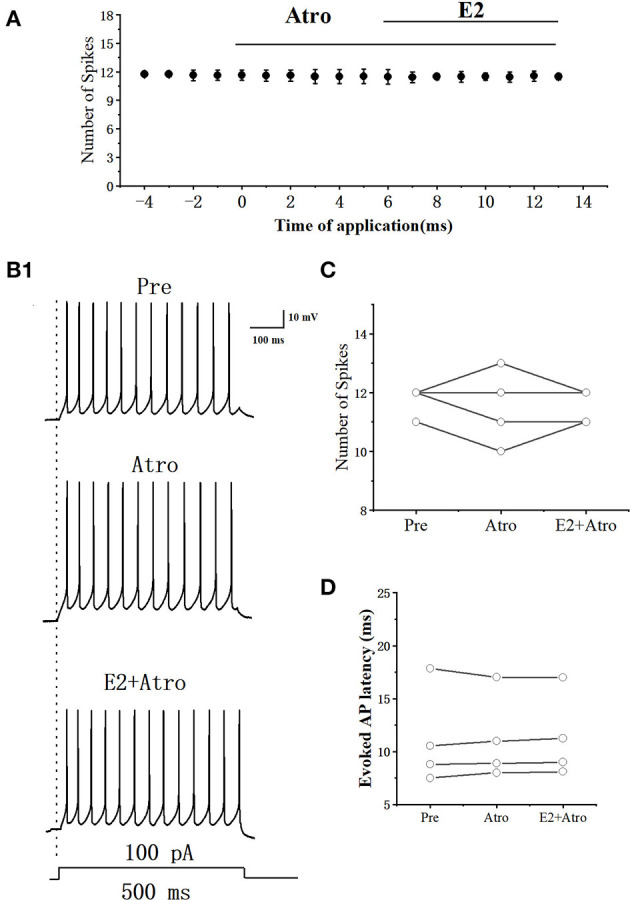
Effect of Atro (10 μM) and E2 (100 pM) + Atropine (10 μM) on the excitability of RA PNs. **(A)** Time course of the number of evoked spikes in the presence of Atro and E2 + Atro (*n* = 4). The line at the top indicates the Atro and E2 + Atro were present in the bath. **(B1)** Example traces from the experiment shown in **(A)**. **(C)** Statistical scatterplot of the number of evoked spikes in the presence of Atro and E2 + Atro (*n* = 4). **(D)** Statistical scatterplot of evoked AP latency in the presence of Atro and E2 +Atro (*n* = 4).

E2 significantly increased the acetylcholine (ACh) release in rats (Gibbs et al., 2014). Whether E2 decreased the excitability of RA PNs by ACh was further tested. As can be seen in Figures 11A, B, we tested the actions of E2 combined mAChR antagonist-atropine (Atro) on the evoked AP firing by 100 pA at 500 ms duration. As shown in Figure 11C, Atropine, E2, and atropine had no change in the number of evoked APs (Pre: 11.75 ± 0.29; Atro: 11.55 ± 0.75; *P* = 0.64, *t* = 0.52; E2+Atro: 11.55 ± 0.33; *n* = 4; *P* = 0.39, *t* = 1, Figure 11C). Atropine, E2, and atropine had no effect on the evoked AP latency (Pre: 11.17 ± 2.67 ms; Atro: 11.23 ± 2.34 ms; *P* = 0.85, *t* = −0.20, E2 + Atro: 11.34 ± 2.31 ms; *n* = 4; *P* = 0.15, *t* =−1.94; Figure 11D). These indicated that the mAChR antagonists blocked the effect of E2 on the excitability of RA PNs.

## Discussion

The findings of this study demonstrated that E2 rapidly suppressed the membrane excitability of RA PNs in zebra finches, which was indicated by a decrease in the spontaneous and evoked spike firing (Figures 1, 2), hyperpolarization of resting membrane potential (Figure 2), increase in the evoked AP latency and AHP time-to-peak, and decrease in the membrane input resistance (Figure 3). The collective impact of these changes was a profound decrease in the overall excitability of RA PNs. The activation of GPER mimicked the effect of E2 (Figures 4–6). G15 and G15 + E2 had no effect on the excitability of RA PNs (Figures 7, 8). G15 blocked the effect of G1 on RA PNs (Figures 9, 10). These results suggested that the E2-binding GPER inhibited the excitability of RA PNs. These findings demonstrated for the first time the role of E2 in modulating the excitability of RA PNs.

In our previous study, castration (low testosterone level) decreased the evoked AP firing rates of RA PNs in male zebra finches (Wang et al., 2014), and this study showed that E2 decreased the membrane excitability of RA PNs. E2 and testosterone as sex steroids induced contrary effects in modulating the membrane excitability of RA PNs in zebra finches. E2 acted as a neurotransmitter *via* binding cell membrane receptors to GPER to acutely change the excitability of RA PNs (Balthazart and Ball, 2006; Zhang et al., 2021), but testosterone took a long time to regulate the membrane excitability of RA PNs *via* conventional genetic mechanisms. Hence, E2 might regulate the excitability of RA PNs in hormones.

The application of E2 decreased the excitability of RA PNs in zebra finches, which was consistent with the result of a recent study on the higher-order auditory region, caudomedial nidopallium (NCM) in songbirds (Scarpa et al., 2022) and nucleus accumbens core in female rats, where E2 decreased the excitability of neurons (Proano and Meitzen, 2020). A potent aromatase inhibitor fadrozole rapidly reduced song acoustic stereotypy (Alward et al., 2016). It happened because fadrozole decreased the level of E2 in the brain, which led to an increase in the excitability of RA PNs and RA receiving more input from LMAN (the lateral portion of the magnocellular nucleus of the anterior neostriatum). The LMAN–RA pathway contributed to generating the variable song (McDonald and Kirn, 2012; Moorman et al., 2021), which was evidence of how fadrozole rapidly reduced song acoustic stereotypy.

Centrally synthesized E2 acts as both a neuroprotective and an anti-inflammatory agent in the brain of songbirds (Pedersen et al., 2016; Saldanha, 2020). The membrane potential is depolarized after ischemia in hippocampal CA1 pyramidal neurons of rats (Isagai et al., 1999; Tanaka et al., 1999), and the excitability of neonatal mouse hippocampus increases *in vitro* after ischemia (Zanelli et al., 2015). In the present study, E2 hyperpolarized membrane excitability in RA PNs of zebra finches, indicating that E2 might have a neuroprotective effect when RA PNs were damaged.

E2 and the agonist of GPER significantly increased the ACh release in the hippocampus of rats (Gibbs et al., 2014). Our previous studies demonstrated that ACh receptor agonist carbachol reduced the excitability of RA PNs by hyperpolarizing the membrane potential (Meng et al., 2016). In our experiment, E2 had no effect on the excitability of RA PNs in the presence of mAChR antagonist Atro (Figure 11), demonstrating that Atro blocked the effect of E2 on the excitability of RA PNs. E2 may cause the release of ACh from local cholinergic terminals and then indirectly reduce the excitability of RA PNs. The estrogen–cholinergic interactions on the excitability of RA PNs will be tested in our future study.

Although G1 mimics the effect of E2 on the excitability of RA PNs, the current–voltage relationship showed reversal potentials of around −70 mV for the E2 exposure and −90 mV for the G1 exposure. E2 and/or G1 cause hyperpolarization and reduced firing by causing an increase in potassium conductance (Dai et al., 2020). The ionic mechanisms involved in modulating the effect of E2 and G1 will be tested in our future study.

The baseline half-width of the APs measured was significantly broader than those measured in other studies (Spiro et al., 1999; Miller et al., 2017; Zemel et al., 2021). The baseline input resistance measured for RA PNs in this study is substantially higher than what has been reported in previous studies (Spiro et al., 1999; Garst-Orozco et al., 2014; Zemel et al., 2021). According to the previous study (Zemel et al., 2021), the temperature (16–20°C) during our experiment may affect the baseline half-width of the APs of RA PNs. The 1.2 mM external calcium was used as the composition of ACSF by previous studies (Spiro et al., 1999; Miller et al., 2017; Zemel et al., 2021), while 2.0 mM external calcium was used as the composition of ACSF in our experiment. We compared the effects of two different extracellular calcium concentrations on the half-width of AP and input impedance of RA PNs (Table 3) and found 1.2 mM external calcium had narrower half-width of AP and lower input impedance compared with 2.0 mM external calcium. In our experiment, the synaptic transmission of RA PNs was not blocked, which may lead to a higher input resistance of RA PNs.

**Table 3:** Comparison of 1.2 mM external calcium with 2.0 mM external calcium to the effects of half-width and membrane input resistance of RA PNs.

**Parameter**	**1.2 mM calcium**	**2.0 mM calcium**	***t*-value*, P*-value**
Half-width (ms)	0.77 ± 0.14 (*n* = 6)	2.06 ± 0.22 (*n =* 11)^**^	*t =* −4.22, *P* < 0.01
Membrane input resistance (MΩ)	198.75 ± 25.46 (*n =* 6)	271.44 ± 18.53^*^ (*n =* 13)	*t* =-2.37, *P* = 0.03

^*^P < 0.05, ^**^P < 0.01.

## Conclusion

The results of this study demonstrated that E2 could acutely inhibit membrane excitability of RA PNs in zebra finches. The E2-binding GPER played a remarkable role in modulating the excitability of RA PNs. This study, for the first time, demonstrated the role of E2 in regulating the membrane excitability of RA PNs.

## Data availability statement

The original contributions presented in the study are included in the article, further inquiries can be directed to the corresponding authors.

## Ethics statement

The animal study was reviewed and approved by the Institutional Animal Care and Use Committee of Jiangxi Science and Technology Normal University (3601020137931).

## Author contributions

YZ and YS performed experiments and prepared the figures. YW, WS, and KZ analyzed the data. SW and WM conceived and designed research. SW prepared the manuscript. All authors contributed to the article and approved the submitted version.
